# The ethical dimension in published animal research in critical care: the public face of science

**DOI:** 10.1186/cc13694

**Published:** 2014-01-14

**Authors:** Meredith Bara, Ari R Joffe

**Affiliations:** Department of Pediatrics, University of Alberta, 4-546 Edmonton Clinic Health Academy; 11405 87 Avenue, Edmonton, AB T6G 1C9 Canada

## Abstract

**Introduction:**

The ethical quality of animal research is important for many reasons, including for maintaining public support. We aimed to determine the reported attention to the ethical dimensions of the 3Rs (Refinement, Reduction, and Replacement) in critical care animal research published in 2012.

**Methods:**

A data-collection form and instruction manual were created based on published recommendations, and completed for all consecutive critical care animal research (using mammals) publications from January to June 2012 in three critical care journals. Predefined subgroups were by journal, sepsis model, and animal age, compared by using the χ^2^ statistic, with statistical significance accepted at *P* < 0.05.

**Results:**

In total, 77 consecutive animal research publications were reviewed. Most studies did not report monitoring the level of anesthesia during invasive procedures, even when muscle paralytics were used, nor monitoring or treatment of expected pain. When euthanasia was used, the method was often not stated, and when stated, most methods were not appropriate for the species. A sample-size calculation was rarely used, and animal numbers were often poorly described. No studies performed a systematic review to ensure that the animal research would be useful and not simple repetition. Seventeen (22%) publications met the composite outcome of, if indicated, using anesthesia and pain control, and stating the method of euthanasia. Most studies were funded with public funds (foundation or government funding). Sepsis models less often met the composite outcome of, if indicated, using anesthesia and pain control, and stating the method of euthanasia (2 (7%) of 27 versus 15 (30%) of 50; *P* = 0.023). No other statistically significant differences were found in reporting of any criterion by animal age, sepsis model, or journal.

**Conclusions:**

Reported (although not necessarily actual) ethical quality of animal research in three high-impact critical care journals during 6 months of 2012 was poor. This has important implications for the practice of critical care animal research.

## Introduction

Biomedical animal research (AR) has an ethical dimension because it can cause harm, including pain, suffering, and early death, to sentient research subjects [1]. To address this ethical dimension, the “3Rs” concept is advocated: Refinement (of experimental methods to minimize harms and maximize benefits), Reduction (of the number of animals used), and Replacement (with alternative methods not using animals) [2]. Previous studies have documented poor methodologic quality of published AR, and poor attention to pain control in published AR; however, none was specifically investigating AR relevant to critical care, nor focused on the ethical dimensions of the 3Rs [3, 4].

Several reasons exist to report and adhere to strict animal-welfare ethical guidelines in performing AR. First, animal pain and distress introduce uncontrolled confounding variables to research, violating the assumption of normal physiology and behavior [1, 5–10]. Pain and distress can impair brain development, cognition, memory, spatial learning, resistance to stress-induced pathology, immunocompetence, disease progression, and behavioral expression [6–9]. Description of and attention to anesthesia, analgesia, and husbandry, including veterinary knowledge, are critical to producing scientifically reliable results [1, 5–10].

Second, consideration of the interests of sentient animals is ethically appropriate, and the methods to monitor for and to treat animal distress should be described to ensure animal welfare [11–14].

Third, poor treatment of experimental animals may lead to loss of public support. Recent surveys suggest that the public places high priority on animal welfare in science [15–17].

The importance of ethical attention to animal welfare has been recognized before. Attempts have been made to address improving animal welfare in intensive care research [10–12, 18, 19]. Calls to improve animal welfare in animal research have occurred in all fields [8, 20–23]. Nevertheless, we are not aware of a review of the quality of reporting of animal welfare in critical care or in other research fields.

Of concern, the literature has shown a poor translation rate of AR to human medicine. This is true in critical care, for example, in the fields of sepsis [24–26], traumatic brain injury [27], resuscitation [28], and spinal cord injury [29], and in other fields, such as stroke [30], asthma [31], and pharmaceutical drug development [32]. Debate continues about whether this is due to methodologic flaws in animal research (for example, lack of randomization, allocation concealment, blinding, and sample-size calculations), or to differences in responses of different complex systems (animals of different species, regardless of methodologic quality) [14, 33–36]. This debate emphasizes the importance that attention to animal welfare plays in limiting any uncontrolled confounding variables in research.

We aimed to determine the reported attention to the ethical dimensions of the 3Rs in critical care AR published during 6 months of the year 2012. Of concern, we found that the reported ethical quality of AR in three high-impact critical care journals during the year 2012 was poor in several areas.

## Materials and methods

We reviewed all consecutive AR published in three prominent critical care journals *(Critical Care Medicine, Intensive Care Medicine, and American Journal of Respiratory and Critical Care Medicine)* during 6 months of the year 2012 to determine the reporting of *a priori* determined ethical-quality factors. These journals were chosen for convenience (they are available at our library) and familiarity (to the authors). No restrictions were made other than the study reported an AR experiment, defined as a procedure for collecting scientific data on the response to an intervention in a systematic way to maximize the chance of answering a question correctly or to provide material for the generation of new hypotheses [5]. A data-collection form and instruction manual were created based on published Canadian, United States, and United Kingdom recommendations for reporting AR [20, 22, 37]. These guidelines were used as they are comprehensive, well referenced, readily available, and based on literature review. The ARRIVE guidelines are endorsed by more than 100 journals from all over the world [20]. Data was obtained for factors important to each of the 3Rs.

The form was completed for all consecutive critical care AR (using mammals) publications from January to June 2012 in the three critical care journals. Both authors independently completed forms for the first 25 articles, discussing the data after every fifth form until consistent agreement was obtained. Thereafter, one author completed forms on all articles, and the other author independently did so for every fourth article (with discussion of the data to maintain consistent agreement), for any data considered uncertain (with discussion until consensus), and for all articles to determine independently the euthanasia method (with discussion until consensus for any disagreement). The University of Alberta Health Research Ethics Board waived the requirement for review because the study involved only publically available data.

The instruction manual made clear definitions for data collection, including the following. Anesthesia was considered indicated for all surgical procedures in all animals having surgery (including tracheostomy, surgical cannulation of vessels, laparotomy, and so on). Pain control was considered reasonably indicated for pain when the animal was conscious; this applies to any pain expected to occur, such as after surgery or burns (as a general guide: would pain be expected if this were a human?) [8]. To determine whether the euthanasia method was appropriate for species, we used the published guidelines reported by the Canadian Council on Animal Care [38], and the American Veterinary Medical Association [39]. These guidelines, based on literature review and veterinary expertise, aim to make death as distress free and painless as possible, by using the most humane method for each species. Some methods are specified as conditionally acceptable if scientific justification is given, as they are associated with distress at the end of life. For example, carbon dioxide, cervical dislocation (without anesthesia), or decapitation are conditionally acceptable in adult rodents, whereas overdose of inhalational anesthetic or intraperitoneal barbiturate with local anesthetic are acceptable. We classified an acceptable method, or a justified conditionally acceptable method as “appropriate” for the species. A sample-size calculation was defined as describing, for the primary outcome, a *P* value (alpha), power (1-beta), and minimally important difference (the difference between groups that the study is powered to detect) [3, 5, 20]. Funding sources, if described were defined as public if from a government or foundation (or charity) grant, and not public, if from industry.

### Statistics

This is an exploratory descriptive study. Data are presented by using descriptive statistics, and were analyzed by using SPSS. Predefined subgroups were by journal, sepsis model, and animal age (neonate, juvenile, adult), compared by using the χ^2^ statistic, with statistical significance accepted at *P* < 0.05, without correction for multiple comparisons. *Post hoc*, we identified another subgroup of rodent/rabbit versus nonrodent/nonrabbit models to determine whether more-advanced species had improved attention to ethical aspects of AR. We also determined three *post hoc* composite outcomes: (a) if indicated, using anesthesia and pain control, and stating the method of euthanasia; (b) the previously mentioned criteria, and in addition that, if stated, the method of euthanasia was appropriate for the species; and (c) the aforementioned criteria, and in addition that a sample-size calculation was provided in the publication.

## Results

Results from review of 77 consecutive AR publications in the three critical care journals are given in Tables 1, 2, and 3.

**Table 1:** **The reported ethical quality of animal research published in three critical care journals during 6 months of 2012: refinement**

Criterion of refinement	Number of 77 publications meeting criterion ( ***n*** (%))
Anesthesia	
Anesthesia was used when indicated^a^	71 (96% of 74)
Monitoring of the level of anesthesia during invasive procedures described	5 (7% of 71)
Muscle paralytics were used during anesthesia	12 (16%)
Monitoring of the level of anesthesia during muscle paralysis described	2 (17% of 12)
Analgesia	
When pain was to be expected, pain medication was used	7 (14% of 49)
Monitoring of the level of pain was stated	2 (4% of 49)
If analgesia was withheld, a justification was stated	0 (0% of 42)
Euthanasia	
When euthanasia was used, the method was stated	38 (59% of 65)
The method of euthanasia was appropriate for that species^b^	15 (39% of 38) [38]
	16 (42% of 38) [39]
Whether survivor animals were euthanized at the end of the experiment was stated when indicated	19 (42% of 45)
Animal husbandry	
Details of animal caging reported^c^	7 (9%)
Any description of room environment^d^	12 (16%)
Any mention of diet^e^	16 (21%)

^a^ In the three cases in which anesthesia was not used, no justification was stated for this.

^b^ Euthanasia appropriate for each species was determined by using the guidelines reported by the Canadian Council on Animal Care [38], and the American Veterinary Medical Association [39], respectively. None of the studies in which methods were not appropriate for species offered a justification.

^c^ Details of caging described were ventilation (1), number per cage (6). No study mentioned sound, air filtering, cage size, handling frequency, bedding material, or cage enrichment.

^d^ Laboratory room environment described was lighting on/off timing (11), temperature (6), and humidity (2). No study mentioned lighting type, noise, or vibration.

^e^ Diet described was food type (3), food access (15; ad lib 12, restricted 3), water type (1), water access (16; all ad lib).

**Table 2:** **The reported ethical quality of animal research published in three critical care journals during 6 months of 2012: reduction and replacement**

Criterion	Number of 77 publications meeting criterion ( ***n*** (%))
**Reduction**	
Sample-size calculation for primary outcome reported	4 (5%)
Animal numbers stated in Methods	61 (79%)
Extra animals mentioned in the Results (that were not stated in Methods)	31 (40%)
**Replacement**	
Systematic review of literature referenced or done	0 (0%)
Alternative methods to using animals were considered^a^	1 (1%)
Why that animal model was chosen was mentioned^b^	17 (22%)

^a^ Alternative not used because “unethical to use in humans.”

^b^ Why that animal model was used: similarity to humans (10), laboratory experience with model (one), a published model (six).

**Table 3:** **The reported ethical quality of animal research published in three critical care journals during 6 months of 2012: composite outcomes and funding sources**

Criterion	Number of 77 publications meeting criterion ( ***n*** (%))
*Composite outcomes*	
If indicated, using anesthesia, pain control, and stating the method of euthanasia	17 (22%)
Criteria above, and the euthanasia method stated was appropriate for the species^a^	8 (10%)
Criteria above, and describing a sample size calculation	3 (4%)
*Funding source (if given):*	
Funded by government	51 (74% of 69)
Funded by foundation	34 (49% of 69)
Funded by industry	11 (16% of 69)

^a^ Euthanasia appropriate for each species determined by using the guidelines reported by the Canadian Council on Animal Care [38].

### Refinement

Attention to reporting of Refinement in AR is shown in Table 1. Few studies reported monitoring the level of anesthesia during invasive procedures (five (7%) of 71), even when muscle paralytics were used (two (17%) of 12), or monitoring or treatment of expected pain (two (4%) of 49, and seven (14%) of 49, respectively). When euthanasia was used, the method was stated for 38 (59%) of 65, and when stated, methods were appropriate for the species for only 15 to 16 (39% to 42%) of 38 [8, 9]. Animal husbandry practices were usually not reported (Table 1).

### Reduction

Attention to reporting of Reduction in AR is shown in Table 2. A sample-size calculation was rarely used (four (5%) of 77). Animal numbers were often poorly described: animal numbers were stated in the methods in 61 (79%) of 77 and extra animals were mentioned in the results that were not stated in the methods in 31 (40%) of 77.

### Replacement

Attention to reporting of Replacement in AR is shown in Table 2. No studies performed a systematic review to ensure that the AR would be useful and not simple repetition. Alternatives to animal models were almost never explicitly considered (one (1%) of 77).

### Composite outcomes

Reporting of the composite outcomes is shown in Table 3. Seventeen (22% of 77) met the composite outcome of, if indicated, using anesthesia and pain control, and stating the method of euthanasia. Eight (10% of 77) met the more-stringent composite outcome that added that the euthanasia method was appropriate for species. Only three (4% of 77) met the most stringent outcome that added that a sample-size calculation was given.

### Funding sources

Reported funding sources are shown in Table 3. Most studies were funded by using public dollars, either from foundations (34 (49%) of 69), or government (51 (74%) of 69) funding agencies.

### Subgroups

Sepsis models less often met the composite outcome of, if indicated, using anesthesia and pain control, and stating the method of euthanasia [2 (7%) of 27 versus 15 (30%) of 50; *P* = 0.023]. No other statistically significant differences were found in reporting of any criterion (in Tables 1, 2, and 3) by age of animal [*n* = 5 neonatal, 1 juvenile, 21 adult], sepsis model [*n* = 27], nor journal [*n* = 49, 19, and 9]. The *post hoc* subgroup of rodent/rabbit versus nonrodent/nonrabbit AR showed some marginally better practices in the nonrodent/nonrabbit animals (Table 4). Improvements included that the method of euthanasia, when reported, was appropriate for the species in eight (62%) of 13), and that extra animals mentioned in the results that were not stated in the methods occurred in only four (17%) of 23. There was also improvement in the composite outcomes of (a) if indicated, using anesthesia, pain control, and stating the method of euthanasia (15 (65%) of 23), and (b) the aforementioned criteria and the euthanasia method was appropriate for the species (11 (48%) of 23).

**Table 4:** **The reported ethical quality of animal research published in three critical care journals during 6 months of 2012: rodent/rabbit versus nonrodent/nonrabbit subgroup**

Criterion	Number of publications meeting criterion ( ***n*** (%))		
	Rodent/rabbit (***n*** = 54)	Nonrodent/nonrabbit (***n*** = 23)	***P*** value
**Refinement**			
When euthanasia was used, the method of euthanasia was stated	25 (50% of 50)	13 (87% of 15)	0.010
This method of euthanasia was appropriate for that species	7 (28% of 25)	8 (62% of 13)	0.003
**Reduction**			
Animal numbers stated in methods	35 (65%)	21 (91%)	0.049
Extra animals mentioned in the results (that were not stated in methods)	27 (50%)	4 (17%)	0.007
**Composite outcomes**			
If indicated, using anesthesia, pain control, and stating the method of euthanasia.	10 (19%)	15 (65%)	<0.001
Criteria above, and the method used was appropriate for the species^a^	5 (9%)	11 (48%)	<0.001
Criteria above, and describing a sample-size calculation	2 (4%)	1 (4%)	ns

Animals in the publications were nonrodent/nonrabbit baboon (1), dog (3), pig (17), sheep (2); rodent/rabbit-mouse (17), rabbit (5), rat (32). *P* value is based on χ^2^ statistics. No other statistically significant differences were noted in any of the other variables shown in Tables 1, 2, and 3.

^a^As there were no differences in 'euthanasia was appropriate for species' in the nonrodent/nonrabbit subgroup by using the two standards (Canadian Council on Animal Care [38], and American Veterinary Medical Association [39]) , only analysis for the Canadian standards is shown.

## Discussion

Reported (although not necessarily actual) ethical quality of AR in three high impact critical care journals during 6 months of 2012 was poor. This is important for several reasons. First, pain and suffering cause changes in physiology, immunology, and behavior that confound interpretation and extrapolation of experimental results [1, 5–10]. Thus, attention to animal welfare is necessary to performing reliable quality research. Second, the interests of sentient animals in avoiding pain and suffering ought to be given more consideration in the reporting of results, reassuring readers that due consideration was given, and of its importance to researchers [11–14]. Third, these publications are, arguably, the public face of science using mostly public funds. Unless the ethical quality of AR reporting improves, AR is at risk of losing public support. Recent surveys suggest that public support for AR is based on the assumption that animal welfare, and attention to the 3Rs more generally, is a priority; public support for AR is far from universal and may be tenuous [15–17].

It is important to point out that it could be argued that what was not reported was actually done. For example, it is possible that monitoring and treatment of postoperative pain was done, but not reported; or that explicit consideration of alternatives to animal models was often done but not reported because of space restrictions. Thus, it is possible that the ethical quality of the AR was good, and only the reporting was poor. Conversely, many of the poor-quality items might have been expected to be reported if they were indeed performed. For example, if a sample-size calculation for a primary outcome, including a *P* value, power, and minimally important difference, were calculated, hence markedly improving the quality of an experimental result, we believe the authors would be likely to report this. A systematic review of the AR in a specific area also, we believe, would be likely to be reported as done, as these are time consuming, and strengthen the importance and validity of a study; few systematic reviews are reported in the AR literature, most of poor quality [40, 41]. Moreover, even when reported, the method of euthanasia used was appropriate for the species in only 39% to 42% of publications [38, 39].

We also argue that better reporting of appropriate monitoring of anesthesia and pain control could only improve the quality, perceived reliability, and public perception of the published AR. For example, if a scoring system to monitor and then treat pain were used, specific criteria were used to titrate anesthesia, or specific enrichment of animal husbandry were used, we believe that space limitations are not a good reason to omit any reference to these high-quality items.

In addition, it is important to report consideration of alternatives to animal models, as these are being developed in new subject fields that animal researchers may not be aware of, and progress in alternative methods requires drawing attention to it [14, 42, 43].

Poor methodologic quality of AR has been reported before [3, 14, 30, 33]. The lack of randomization, allocation concealment, blinding, primary outcome, and sample-size calculation, multiple statistical testing, and publication bias have been assumed to account for the poor translation of AR to human medicine [24–32]. Poor attention to pain control in AR has also been reported before, although infrequently [4, 44, 45]. Pain and distress are major confounding variables in AR because of their effects on physiology, immunity, and behavior [1, 5–10]. The lack of proper attention to pain and distress may also account for the poor translation of AR to human medicine. Our findings significantly add to this literature because previous publications have not focused on the entire spectrum of the ethical dimension of AR, as in this study, nor on critical care AR in particular.

The findings of this study are concerning. The ARRIVE guidelines, supported by many high-impact journals, and other national guidelines, suggest inclusion in publications of many of the factors we found to be poorly reported, including: monitoring of the level of anesthesia, monitoring of pain by using a validated scale, titration of anesthesia and pain control to stated goals, optimal methods of euthanasia, details of animal husbandry, sample-size calculation, clear animal numbers stated in methods and all results, systematic review of the animal literature to justify the current study, and consideration of all possible alternatives to use of animals [5, 8, 20, 22, 23, 37]. Some controversial issues are raised by these suggestions. For example, pain medications, or different methods of euthanasia, can influence the results of an experiment, making it difficult to compare with previously published findings [10, 12, 19]. Sometimes less humane end points (such as mortality) are said to be necessary to test a therapy (such as for sepsis) [10, 19]. However, the counterargument is that pain and distress may confound the study results, and may thus be a reason for the poor translation of AR to humans. In other words, because no novel therapy based on AR has been successful in treatment of sepsis in humans [24–26], researchers should seriously consider whether this is because of lack of attention to pain and distress in the experimental animal subjects. The alternative to this explanation is that responses to interventions are different in different species due to in-principle differences in initial conditions of complex systems (the organism) resulting in different genomic (and hence functional) outcomes [46–51].

Limitations of this study include the small sample size of publications reviewed, the limited scope to critical care AR, and the low power to detect differences between subgroups, particularly given multiple comparisons. Nevertheless, this study is the first to focus on the ethical dimension of AR in critical care, and reviewed a relatively large series of consecutive publications in three high-impact critical care journals by using an objective data-collection form and instruction manual. Whether our findings from this small critical care AR cohort generalize to most AR is unknown; however, we believe this is likely because critical care experiments are more invasive than most other AR.

We believe that it is time for a serious debate about the methods of AR in critical care. Better attention to, and reporting of, ethical factors in AR can only improve the research quality, distress of the animals, and public perception of AR. Journal editors and reviewers, and funding agencies should use their influence to improve quality reporting of AR they publish and support [52, 53].

## Conclusions

We found that reported (although not necessarily actual) ethical quality of AR in three high-impact critical care journals during 6 months of the year 2012 was poor. These findings warrant the attention of clinicians, researchers, journal editors and reviewers, and funding agencies. Improved attention to the 3Rs by these groups can only improve AR quality, animal comfort, and the public perception of AR.

## Key messages

1. Reported ethical quality of animal research in three high-impact critical care journals during 6 months of 2012 was found to be poor.
2. Better reporting of ethical factors in AR may improve the research report and improve public perception of AR.
3. These publications are, arguably, the public face of science using mostly public funds; unless the ethical quality of animal research reporting improves, animal research may be in jeopardy of losing public support.

## References

[1] Rollin BE (2009). Scientific autonomy and the 3Rs. Am J Bioeth.

[2] Whittall H, Nuffield Council on Bioethics (2009). Information on the 3Rs in animal research publications is crucial. Am J Bioeth.

[3] Kilkenny C, Parsons N, Kadyszewski E, Festing MFW, Cuthill IC, Fry D, Hutton J, Altman DG (2009). Survey of the quality of experimental design, statistical analysis and reporting of research using animals. PLoS ONE.

[4] Carbone L (2011). Pain in laboratory animals: the ethical and regulatory imperatives. PLoS ONE.

[5] Festing MF, Altman DG (2002). Guidelines for the design and statistical analysis of experiments using laboratory animals. ILAR J.

[6] Poole T (1997). Happy animals make good science. Lab Animals.

[7] Balcombe JP (2006). Laboratory environments and rodents’ behavioral needs: a review. Lab Animals.

[8] National Research Council Committee on recognition and alleviation of pain in laboratory animals (2009). Recognition and Alleviation of Pain in Laboratory Animals.

[9] Eisenberger N, Cole SW (2012). Social neuroscience and health: neurophysiological mechanisms linking social ties with physical health. Nat Neurosci.

[10] Nemzek JA, Xiao HY, Minard AE, Bolgos GL, Remick DG (2004). Humane endpoints in shock research. Shock.

[11] Galley HF (2010). Mice, men, and medicine. Br J Anaesth.

[12] Huet O, Ramsey D, Miljavec S, Jenney A, Aubron C, Aprico A, Stefanovic N, Balkau B, Head GA, de Haan JB, Chin-Dusting JPF (2013). Ensuring animal welfare while meeting scientific aims using a murine pneumonia model of septic shock. Shock.

[13] Rollin BE (2007). Animal research: a moral science. EMBO Rep.

[14] Ferdowsian HR, Beck N (2011). Ethical and scientific considerations regarding animal testing and research. PLoS ONE.

[15] Kmietowicz Z (2012). Researchers promise to be more open about use of animals in their work. BMJ.

[16] Goodman J (2012). Public opinion on animal testing. AV Magazine.

[17] Goodman JR, Borch CA, Cherry E (2012). Mounting opposition to vivisection. Contexts.

[18] Morton DB, Briffiths PH (1985). Guidelines on the recognition of pain, distress and discomfort in experimental animals and an hypothesis for assessment. Veterinary Record.

[19] Nemzek JA, Hugunin KMS, Opp MR (2008). Modeling sepsis in the laboratory: merging sound science with animal well-being. Comp Med.

[20] Kilkenny C, Browne WJ, Cuthill IC, Emerson M, Altman DG (2010). Improving bioscience research reporting: the ARRIVE guidelines for reporting animal research. PLoS Biol.

[21] Canadian Council on Animal Care in Science (1998). CCAC Guidelines on: Choosing an Appropriate Endpoint in Experiments Using Animals for Research, Teaching and Testing.

[22] Canadian Council on Animal Care in Science (1997). CCAC Guidelines on: Animal Use Protocol Review.

[23] National Research Council Committee on Recognition and Alleviation of Distress in Laboratory Animals (2008). Recognition and Alleviation of Distress in Laboratory Animals.

[24] Dyson A, Singer M (2009). Animal models of sepsis: why does preclinical efficacy fail to translate to the clinical setting. Crit Care Med.

[25] Opal SM, Patrozou E (2009). Translational research in the development of novel sepsis therapeutics: logical deductive reasoning or mission impossible?. Crit Care Med.

[26] Marshall JC, Deitch E, Moldawer LL, Opal S, Redl H, van der Poll T (2005). Preclinical models of shock and sepsis: what can they tell us?. Shock.

[27] Xiong Y, Mahmood A, Chopp M (2013). Animal models of traumatic brain injury. Nat Rev Neurosci.

[28] Reynolds PS (2012). Twenty years after: do animal trials inform clinical resuscitation research. Resuscitation.

[29] Akhtar AZ, Pippin JJ, Sandusky CB (2009). Animal models of spinal cord injury: a review. Rev Neurosci.

[30] Sena E, van der Worp B, Howells D, Macleod M (2007). How can we improve the preclinical development of drugs for stroke?. Trends Neurosci.

[31] Holmes AM, Solari R, Holgate ST (2011). Animal models of asthma: value, limitations and opportunities for alternative approaches. Drug Discov Today.

[32] Pammolli F, Magazzini L, Riccaboni M (2011). The productivity crisis in pharmaceutical R&D. Nat Rev Drug Discovery.

[33] Van der Worp HB, Howells DW, Sena ES, Porritt MJ, Rewell S, O’Collins V, Macleod MR (2010). Can animal models of disease reliably inform human studies?. PLoS Med.

[34] Haouzi P (2011). Murine models in critical care research. Crit Care Med.

[35] Horrobin DF (2003). Modern biomedical research: an internally self-consistent universe with little contact with medical reality?. Nature Rev Drug Discovery.

[36] Schultz MJ, van der Poll T (2002). Animal and human models for sepsis. Ann Med.

[37] Institute for Laboratory Animal Research, National Research Council (2011). Guidance for the Description of Animal Research in Scientific Publications.

[38] Canadian Council on Animal Care in Science (2010). CCAC Guidelines on: Euthanasia of Animals Used in Science.

[39] American Veterinary Medical Association (2007). AVMA Guidelines on Euthanasia.

[40] Lamontagne F, Briel M, Duffett M, Fox-Robichaud A, Cook DJ, Guyatt G, Lesur O, Meade MO (2010). Systematic review of reviews including animal studies addressing therapeutic interventions for sepsis. Crit Care Med.

[41] Mignini LE, Khan KS (2006). Methodological quality of systematic reviews of animal studies: a survey of reviews of basic research. BMC Med Res Methodol.

[42] Russell D (2012). Why animal ethics committees don’t work. Between Species.

[43] Littman BH, Williams SA (2005). The ultimate model organism: progress in experimental medicine. Nat Rev Drug Discovery.

[44] Flecknell P (2005). Partnerships for progress. Vet Anaesth Analgesia.

[45] Fenwick N, Danielson P, Griffin G (2011). Survey of Canadian animal-based researchers’ views on the three Rs: replacement, reduction and refinement. PLoS ONE.

[46] Shanks N, Greek R, Greek J (2009). Are animal models predictive for humans?. Phil Ethics Humanities Med.

[47] West GB (2012). The importance of quantitative systemic thinking in medicine. Lancet.

[48] Seok J, Warren S, Cuenca AG, Mindrinos MN, Baker HV, Xu W, Richards DR, McDonald-Smith GP, Gao H, Hennessy L, Finnerty CC, Lopez CM, Honari S, Moore EE, Minei JP, Cuschieri J, Bankey PE, Johnson JL, Sperry J, Nathens AB, Billiar TR, West MA, Geschke MG, Klein MB, Gamelli RL, Gibran NS, Brownstein BH, Miller-Graziano C, Calvano SE, Mason PH (2013). Genomic responses in mouse models poorly mimic human inflammatory diseases. Proc Natl Acad Sci U S A.

[49] Odom DT, Dowell RD, Jacobsen ES, Gordon W, MacIsaac KD, Rolfe PA, Conboy CM, Gifford DK, Fraenkel E (2007). Tissue-specific transcriptional regulation has diverged significantly between human and mouse. Nat Genetics.

[50] Romero IG, Ruvinsky I, Gilad Y (2012). Comparative studies of gene expression and the evolution of gene regulation. Nature Rev Genetics.

[51] Brawand D, Soumillon M, Necsulea A, Julien P, Csardi G, Harrigan P, Weier M, Liechti A, Aximu-Petri A, Kircher M, Alberta FW, Zeller U, Khaitovich P, Grutzner F, Bergmann S, Nielsen R, Paabo S, Kaessmann H (2011). The evolution of gene expression levels in mammalian organs. Nature.

[52] Osborne NJ, Payne D, Newman ML (2009). Journal editorial policies, animal welfare, and the 3Rs. Am J Bioethics.

[53] Marusic A (2009). Can journal editors police animal welfare?. Am J Bioethics.

